# Higher Mixed lineage Kinase Domain-like protein (MLKL) is associated with worst overall survival in adult-type diffuse glioma patients

**DOI:** 10.1371/journal.pone.0291019

**Published:** 2023-08-31

**Authors:** Guilherme Afonso Vergara, Gisele Cristine Eugenio, Suzana Maria Fleury Malheiros, Elivane da Silva Victor, Ricardo Weinlich

**Affiliations:** 1 Hospital Israelita Albert Einstein, São Paulo, SP, Brazil; 2 Departamento de Neurologia/Neurocirurgia, Universidade Federal de São Paulo, São Paulo, SP, Brazil; All India Institute of Medical Sciences, INDIA

## Abstract

**Introduction:**

Recently, the search for novel molecular markers in adult-type diffuse gliomas has grown substantially, yet with few novel breakthroughs. As the presence of a necrotic center is a differential diagnosis for more aggressive entities, we hypothesized that genes involved in necroptosis may play a role in tumor progression.

**Aim:**

Given that MLKL is the executioner of the necroptotic pathway, we evaluated whether this gene would help to predict prognosis of adult gliomas patients.

**Methods:**

We analyzed a publicly available retrospective cohort (n = 530) with Kaplan Meier survival analysis (p<0.0001) and both uni- and multivariate Cox regression models.

**Results:**

We determined that MLKL is an independent predictive prognostic marker for overall survival in these patients (HR: 2.56, p<0.001), even when controlled by the CNS5 gold-standard markers, namely IDH mutation and 1p/19q Codeletion (HR: 1.68, p = 0.013). These findings were confirmed in a validation cohort (n = 325), using the same cutoff value. Interestingly, higher expression of MLKL is associated with worse clinical outcome for adult-type diffuse glioma patients, which is opposite to what was found in other cell cancer types, suggesting that necroptosis undertakes an atypical detrimental role in glioma progression.

## Introduction

Adult-type diffuse gliomas are the most common central nervous system (CNS) cancer in adults and comprise a clinically distinct group of tumors. The use of molecular markers to improve the clinical management of patients is a growing field in several diseases, especially in cancer. Regarding CNS tumors, this significantly improved overall patient prognostic assessment, as it enhanced the glioma classification system, grouping patients with similar biological behaviors. For diffuse gliomas, the 2016 update of the WHO CNS tumor classification guidelines [1], along with the cIMPACT-NOW consortium [2], acknowledged integrated diagnosis criteria as the gold-standard type-defining system, further refined by the new WHO 5^th^ edition system of CNS tumors classification (CNS5) [3]. This approach combines classical histopathological analysis with molecular markers to subgroup patients into more biologically and clinically homogeneous group, for optimal management.

The main molecular markers used for adult-type diffuse glioma diagnosis to date are the mutational status of isocitrate dehydrogenase (NADP^+^) 1 (IDH1) or 2 (IDH2) [4, 5] and deletion of both the short arm of chromosome 1 and the long arm of chromosome 19 (1p/19q codel) [6, 7]. Noteworthy, the 1p/19q codel only occurs in patients harboring mutation in either IDH1 or IDH2 (herein named as IDHm) [8], ultimately subgrouping glioma patients into three molecularly distinct groups: 1) tumors harboring IDHm with 1p/19q codel (Oligodendroglioma), 2) tumors with IDHm and intact 1p/19q (Astrocytoma), and 3) tumors with IDH wild-type (Glioblastoma) [3, 4]. It is important to highlight that, until CNS5 publication, tumors in the latter group that didn’t present proliferative microvasculature or necrosis were classified as Astrocytoma, IDH wild-type grade 2 or 3. Together with the Astrocytomas (IDHm) and Oligodendrogliomas, they were collectively called Lower Grade Gliomas (LGG) [1].

Despite the growing number of markers and updates on CNS classification, LGG patients’ outcome still cannot be fully predicted. Therefore, novel candidates to either replace or enrich current classifications are one of the main focuses of research in the field. Cell death pathways have already rendered the identification of promising predictive markers, potential therapeutic targets and relevant insights concerning tumor development and progression [9–11]. Regarding gliomas, however, these pathways are yet to be explored as a source of novel molecular markers.

Necroptosis is a particularly interesting pathway, as it is a pro-inflammatory regulated cell death pathway, which can elicit a robust immune response and promote angiogenesis [12, 13]. It can be induced by death receptors ligation, TRIF-mediated toll-like receptor signaling, DAI/ZBP-1-mediated viral sensing as well as by type-1 interferon signaling [12, 14, 15]. These stimuli induces the auto-phosphorilation of Receptor-interacting serine/threonine-protein kinase 3 (RIPK3) that, in turn, phosphorilates the Mixed-lineage Kinase Domain like protein (MLKL), the main effector of the necroptotic pathway [16]. Once phosphorilated, MLKL proteins change their conformational structure, oligomerize and insert in the plasma membrane, acting as a destabilizer and inducing its rupture, causing the main phenotype of the necrotic cell death, which is the loss of membrane integrity [17–20].

Necroptotic molecules, such as RIPK3 and MLKL, were already reported as prognostic markers in many tumor types. High expression of RIPK3 is associated with longer overall survival and lower risk of disease progression in colon cancer [21]. Higher RIPK3 expression is also associated with decreased risk of metastasis and disease progression in breast cancer patients [22]. MLKL can be used not only as a progression-free marker in pancreatic adenocarcinoma, but also as an overall survival marker in ovarian cancer [23]. In all cases above, higher expression is translated into more favorable outcomes, probably due to increased sensitivity to the necroptotic pathway and, possibly, higher rates of immunogenic cell death and increased immune response [24].

Gliomas, however, develop in the CNS, a region in which the immune response are actively suppressed and very limited, to avoid devastating tissue damage [25, 26]. In this context, our hipothesis is that increased susceptibility to necroptosis and its pro-inflammatory properties could favor an opposite, pro-tumorigenic outcome. Indeed, it was previously shown that higher RIPK3 expression is associated with worst overall survival in LGG patients [27].

Here, we demonstrate that MLKL act as an independent prognostic biomarker in LGG similarly to RIPK3 and supports the rationale that increased tumor expression of necroptotic molecules is detrimental to these patients.

## Materials and methods

### Molecular & clinical data

RNAseq data of LGG samples were downloaded from the cBioPortal online platform [28, 29]. We filtered results from the ‘Brain Lower Grade Glioma (TCGA, Provisional) dataset [4], composed of 530 samples for our discovery cohort. For the validation cohort, we downloaded RNAseq data from the Chinese Glioma Genome Atlas (CGGA) [30, 31], containing 325 glioma samples, from which 142 are diagnosed as LGG (batch 1–325- and 2–693 -from CGGA portal). Clinical data were available for each sample in the same platforms. For the discovery cohort, reference papers confirmed that both IDH1 and IDH2 data are lumped together as IDH mutation status variable (as available in the public portal and used in our analysis). Regarding the validation dataset, since we could not confirm whether IDH mutation status variable considers both IDH1 and IDH2 or only one of them, we kept the original designation of ‘IDH mutation status’. The inclusion criteria comprised (I) ≥18 years of age and (II) a diffuse lower grade glioma diagnosis. The exclusion criteria encompassed missing data on (I) MLKL expression or (II) Overall Survival time/status, (III) relapse/secundary tumor data-only. The final compositions from both cohorts included samples of primary tumors derived from both male and female adult (age ≥18 years old) patients, with a total of 513 and 134 samples, respectively for the discovery and validation datasets. As only retrospective data was used, there was no randomization, blinding or power analysis performed prior to the formal analysis deemed irrelevant.

To standardize the RNAseq data from two different platforms, we opted for a z-score conversion, to conserve the original non-parametrical distribution of the data, as accused by box-plot and histogram analysis, as well as Shapiro-Wilk’s normality test (p<0.05). In the discovery set, we filtered all the population diploid for MLKL (also available at cBioPortal website) to compose a reference population. In the validation cohort, since no information regarding ploidy were available at CGGA’s portal, we considered all the 134 samples as reference population, in order to minimize any bias toward reference selection.

### Statistical analysis

To check whether MLKL can act as a prognostic marker, we chose overall survival as clinical outcome and conducted Kaplan Meier survival analysis with LogRank comparison tests followed by Cox regression modeling, both in univariate and multivariate approaches.

To categorize patients into two groups, we calculated the optimal cutoff value through the minimum p-value, with bootstrap validation for inferential interpretation. Furthermore, since all statistical approaches that rely on multiple testing/sampling are prone to biases, we corrected the p-value (Lausen & Shumacher method) [32] and the Hazard Ratio estimations (heuristic contraction factor) accordingly. Additionally, since the distribution is not normal, we did all testing and description techniques according to non-parametric principles (Mann-Whitney testing for comparison between two groups, Kruskall-Wallis when comparing three or more groups and descriptive statistics for numeric variables as median and interquartile range).

Our strategy focused in a more tailored selection for multivariate assessment of MLKL’s predictive power and reliability. Firstly, the selection was made based on the univariate regression models, assuming a significance α = 1% (p-value<0.01), to have a thinner refinement of all possible candidates that are significantly related to overall survival and with high sample size (given that many variables have NA entries, the statistical power/validity of analysis could be flawed). For all subsequent analysis, we assumed the standard α = 5% (p-value<0.05). Secondly, we checked for correlation between the filtered variables for optimal multivariate adjustment, since using a selection method could jeopardize any possible associations due to multicollinearity.

### Ethical approval

All the data gathered is available in the aforementioned repository platforms. All patients signed an informed consent for publishing their clinical and molecular data in each original study beforehand and in all samples, they are unidentifiable with ID codes for reference, remaining in secrecy.

## Results

Given that CNS5 publication is quite recent, the glioma public datasets lack important data that are needed to fully reconstitute this classification system. In fact, the most comprehensive cohort available was structured only with LGG samples. Therefore, we have opted to use the molecular criteria established by the 2016 WHO updated 4^th^ Edition system as well as the histological type and grade criteria available in the dataset.

To investigate whether MLKL could act as overall survival prognostic marker in LGG patients, we first described all available explanatory variables in the discovery cohort (Table 1 and S1 Table in S1 File).

**Table 1:** Description of the main variables available for analysis. The cohort used as discovery set is from the TCGA Firehose Legacy study of ‘Lower Grade Glioma’, available at cBioPortal online platform.

Variable	O.S. Status		Total
	Alive	Dead	
MLKL			
Median (1^st^ Q; 3^rd^ Q)	-0.28 (-0.63; 0.18)	-0.02 (-0.62; 0.63)	-0.23 (-0.63; 0.31)
Age at Diagnosis			
Median (1^st^ Q; 3^rd^ Q)	39 (32; 51)	46 (35; 60)	41 (33; 53)
IDH Mutation			
No	44 (11.4%)	50 (40.3%)	94 (18.4%)
Yes	342 (88.6%)	74 (59.7%)	416 (81.6%)
1p/19q Codeletion			
No	241 (62.3%)	104 (82.5%)	345 (67.3%)
Yes	146 (37.7%)	22 (17.5%)	168 (32.7%)
IDH and 1p/19q status			
IDH Wildtype	44 (11.4%)	50 (40.3%)	94 (18.4%)
IDH Mutated and 1p/19q intact	146 (37.8%)	22 (17.8%)	168 (32.9%)
IDH mutated and 1p/19q codeleted	196 (50.8%)	52 (41.9%)	248 (48.6%)

Q: Quartile. Numerical data described as non-parametrical data, considering median and interquartile range. Categorical data described with number of samples and percentile.

Next, we checked which variables were associated with survival status by performing a univariate Cox regression model, considering overall survival (O.S.) as clinical outcome for each variable. The results of the main candidates are compiled in Table 2 and additional variables tested are shown in S2 Table in S1 File.

**Table 2:** Univariate model of the main explanatory variables used for molecular and histological diagnosis. See other models in S2 Table in S1 File.

Variables	N (%)	HR (95% IC)	p-value
MLKL*	512 (100)	1.03 (1.02, 1.04)	<0.001
Age at Diagnosis	512 (100)	1.06 (1.04, 1.07)	<0.001
IDH Mutation			
Yes	415 (81.5)	Ref.	
No	94 (18.5)	6.49 (4.46, 9.43)	0.001
1p/19q Codeletion			
No	345 (6,4)	Ref.	
Yes	167 (32,6)	0.388 (0.243, 0.621)	<0.001
IDH and 1p/19q Status			
IDH mutated and 1p/19q codeleted	94 (18,5)	Ref.	
IDH mutated and 1p/19q intact	248 (48,7)	1.540 (0.927, 2.557)	0.096
IDH wildtype	167 (32,8)	8.617 (5.134, 14.463)	<0.001

N: sample size; HR: Hazard ratio; CI: Confidence Interval; *Consider 0.1 as unit of variation in the z-score scale for interpretation of MLKL HR estimation.

We confirmed that the “Age at Diagnosis”, “IDH mutational status” and “1p/19q codel” main variables do affect patients’ prognosis (all with p<0.001), as expected. Interestingly, MLKL expression level also presents itself as a prognostic factor for LGG patients (p<0.001), whereas for each 0.1 increase in MLKL expression Z-score, the risk of death for this patient increases by 3%.

Considering a more dynamic and feasible clinical approach for MLKL expression levels, a standard reference value for comparison should be established. In this sense, to group patients into high and low MLKL expression groups, we elected an optimal cut-off z-score value of 0.439 through minimum p-value approach with bootstrap validation. Regarding O.S., high MLKL expression patients present an increased risk of death (HR: 2.56 (1.75, 3.75), p<0.001), suggesting that poorer outcomes are associated with higher expression.

**Fig 1:**
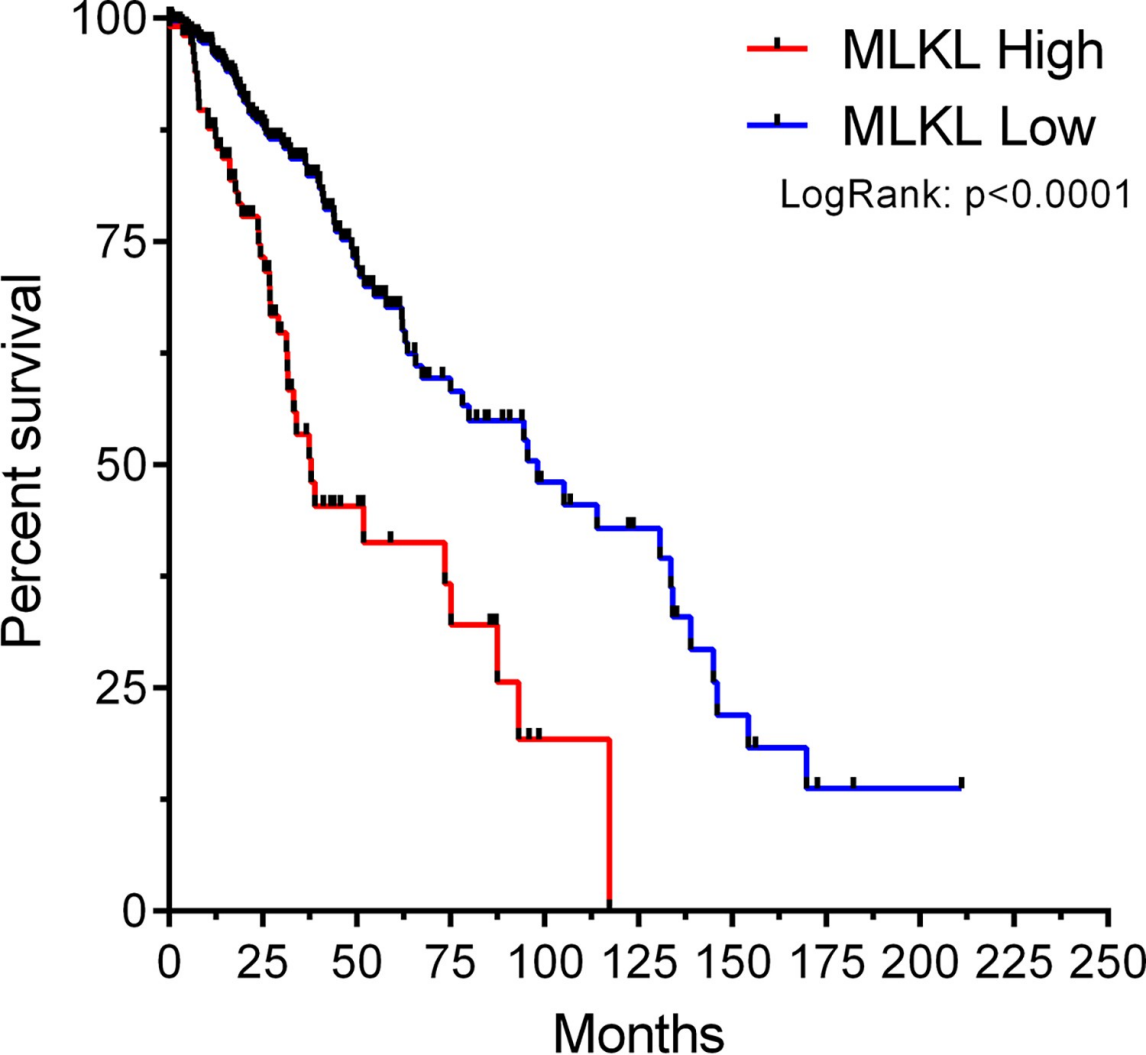
Kaplan Meier survival analysis of lower grade glioma patients considering the expression of MLKL. We grouped patients into high (n = 109 red, dotted) or low (n = 400 blue, solid) expression levels, according to the optimal cut-off value. For inferential interpretation, we performed LogRank tests.

Even when categorized, MLKL retained prognostic factor traits with statistical significance, successfully splitting patients into two clinical distinct groups regarding overall survival (<0.001). Our analysis suggests that patients with high MLKL expression levels have a poorer prognosis, with an estimated 156% increased risk of death. The Kaplan Meier survival curve confirming these findings are presented in Fig 1, with a median survival of 37.84 and 98.16 months for high and low expression groups, respectively.

**Fig 2:**
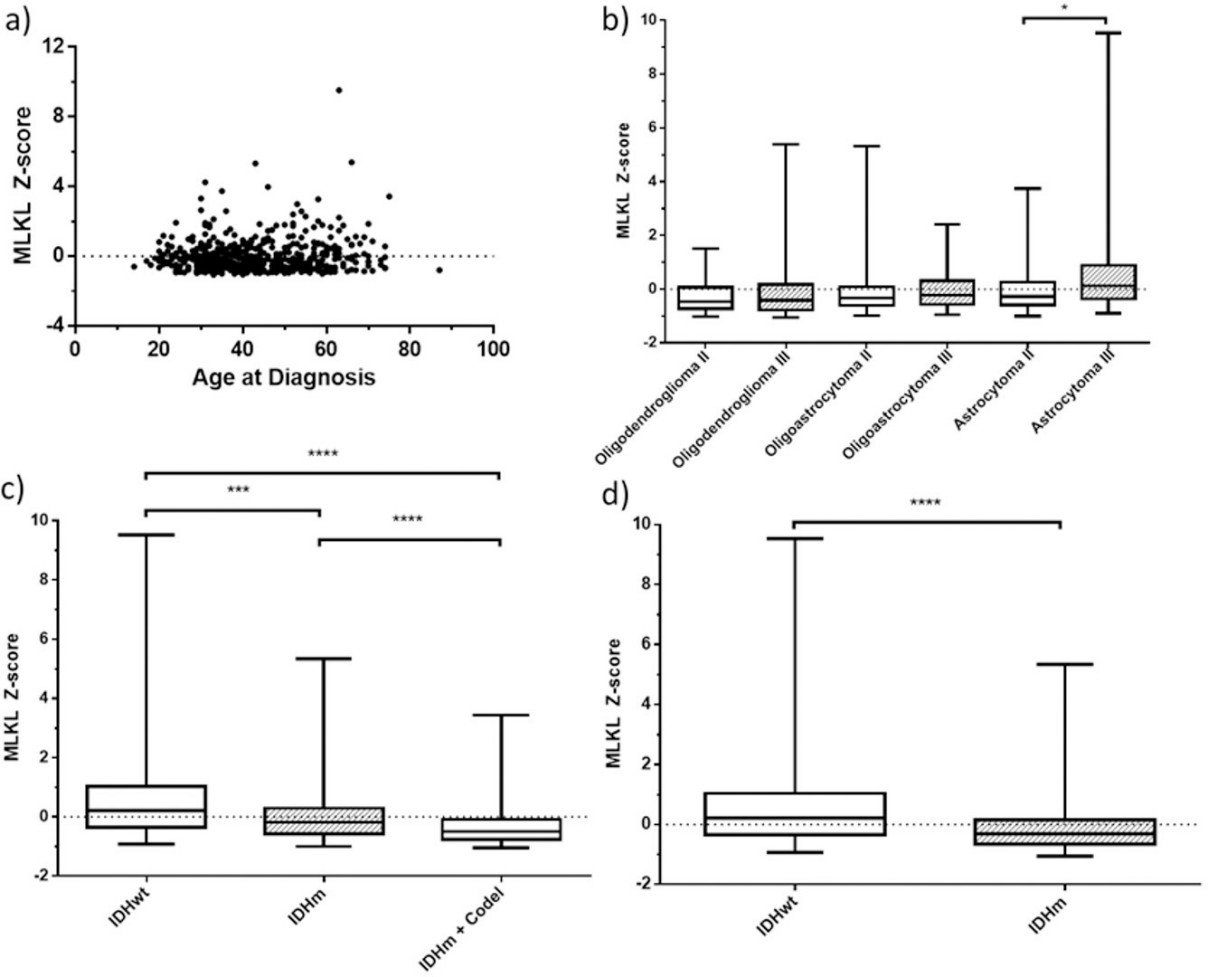
Assessment of MLKL’s association with known clinical variables. Using continuous data of MLKL’s expression (n = 512), we checked for (A) correlation between MLKL and Age at diagnosis and association between MLKL expression and both (B) histological diagnostic, following grading and type from the available dataset (WHO 2016) and (C) molecular diagnostic groups as well as the (D) main prognostic marker IDH mutational status. A: Astrocytoma; O: Oligodendroglioma; OA: Oligoastrocytoma (outdated). Mann-Whitney test was used to estimate the p-value when comparing two groups. Kruskall-Wallis was used to estimate the p-value when comparing three or more groups.

To assess whether MLKL is associated with the main clinically relevant variables, we checked MLKL expression correlation with age at diagnosis and both molecular and histological gold-standards for diagnosis. We observed that there is no association between age and MLKL expression (Fig 2A, Spearman’s rho = 0.034), showing no trends of different MLKL expression profile across the study population. Considering the histological diagnosis (Fig 2B), only Astrocytomas comprise patients with significantly distinct MLKL expression profiles, being higher in grade 3 subtype, the most aggressive form of LGGs. All molecularly defined diagnostic groups presented significantly different levels of MLKL expression (Fig 2C), being higher in the IDH wild-type group; composed of patients with the least favorable outcome. Given that 1p/19q codel may be used only as a diagnostic but not an independent O.S. prognostic marker [4, 27], we also analyzed MLKL expression levels association with IDH mutational status alone (Fig 2D). Distinct MLKL expression levels are also associated with IDH mutational status, following the same trend, as higher expression levels are more prevalent in IDH wild-type group.

Since both histological and the molecular diagnostic criteria concerns the same clinical outcome (disease severity), we opted to analyze two separate multivariate models, one using the classical histopathological (type and grade) and the other with the molecular criteria (IDH and 1p/19q status) (Table 3 and S3 Table in S1 File). In both settings, we demonstrated that MLKL expression can be used as an independent prognostic marker regardless of diagnostic criteria used (p = 0.002 and p = 0.013, for histological and molecular criteria, respectively).

**Table 3:** Multivariate model of MLKL expression controlled by IDH mutation/codeletion status and age at diagnosis.

Variables	N	HR	95% CI for HR		p-value
			LL	UL	
IDH mutation and codeletion					
Mutation and positive codeletion	167	Ref.			
Mutation and negative codeletion	248	2.06	1.22	3.47	0.007
No mutation	94	6.63	3.83	11.48	<0.001
MLKL					
Low	400	Ref.			
High	109	1.68	1.12	2.54	0.013
Diagnostic age	509	1.05	1.04	1.07	<0.001

N: sample size; HR: Hazard ratio; LL: Lower limit; UL: Upper limit; CI: Confidence Interval; Ref.: Reference. Considering “No Mutation” as reference, “Mutation and negative codeletion” have a 69% lower risk of death (HR (CI95%): 0.31 (0.20–0.49)), with p<0.001.

To confirm such findings, we also performed a multivariate analysis with the data available in the validation cohort (n = 134). However, as 1p/19q codel information was not available, the analysis was done using “MLKL expression”, “age at diagnosis” and “IDH mutational status” alone (see S4 Table in S2 File for descriptive statistics and S5 Table in S2 File for univariate models for age at diagnosis). MLKL expression (continuous) and IDH mutational status; confirming it as a prognostic factor. To further validate the cut-off value established in the discovery cohort, we grouped patients in the validation cohort based on the same value (S4 Table in S2 File and S1 Fig in S2 File), successfully separating patients into two clinically distinct groups, regarding O.S. (with HR of 2.64). Furthermore, we also assessed MLKL expression regarding the major clinical variables, whereas only IDH mutational status is associated with its expression (S2c Fig in S2 File). Ultimately, even when controlled by IDH status and age, MLKL expression trends towards a prognostic factor role in LGG patients’ O.S., being higher in worst cases (HR: 1.96), confirming our previous findings (S7 Table in S2 File). Taking together, our results strongly suggests that MLKL is a remarkable independent prognostic marker.

## Discussion

The recent fast-paced expansion of our knowledge about the molecular pathways involved in the pathogenesis of the different cancer entities enabled the discovery of multiple genetic and epigenetic biomarkers that have been already successfully implemented in the clinical setting. They have been quite valuable to subgroup cancer patients with more homogeneous clinical outcomes, such as response to treatment, disease-free survival time (DFS), progression-free survival time (PFS) and overall survival time (O.S.).

Two molecular biomarkers–IDH mutational status and 1p/19q codeletion–were a major breakthrough to establish an integrated diagnosis criteria for diffuse gliomas, and were included in the 2016 WHO updated 4^th^ Edition on CNS tumor classification [1]. This classification system was considered a major advance over the traditional criteria based only in morphology/histology and underscores the growing relevance of these markers for patient management. Importantly, in the 2021 CNS5, IDH and 1p/19q were maintained as the central entity-defining biomarkers for adult-type diffuse gliomas. Additional biomarkers, such as CDKN2A/B homozygous deletion, ATRX loss-of-expression, TERT promoter mutation, EGFR gene amplification and +7/-10 chromosome copy number changes, were officialy included in the CNS5 classification system to help designating glioma subtypes with more homogeneous clinical behaviors [3, 33, 34]. However, despite using the most up-to-date criteria, diffuse glioma patients within the same subtype can still present very dissimilar clinical outcomes, indicating the necessity of an improved classification system that could more accurately predict patient prognosis and response to treatment.

Resistance to apoptosis is one of the major hallmarks of cancer and the expression levels of several members of this pathway have been successfully used as diagnostic, prognostic and treatment response predictors in numerous cancers [35]. In the past two decades, novel cell death modes, such as pyroptosis, necroptosis and ferroptosis, were described. Contrary to apoptosis, they present necrotic-like features that can induce pro-inflammatory and immunogenic responses. Although still scarcely explored, there is already appealing evidence that their expression levels can be used as relevant clinical biomarkers [36–38].

In this paper, we demonstrated that MLKL expression levels, a key element of the necroptotic pathway, is an independent overall survival biomarker, even when controlled by the main clinically useful variables available in the current datasets and regardless of whether histological or integrated diagnostic criteria are used. In other words, considering two glioma patients with same age and molecular or histological diagnostics yet with distinct overall survival, the difference in MLKL expression level contributes to explain the observed discrepancy in this clinical outcome. Therefore, MLKL quantitation could be used to better refine prognostic group projections either in combination with the existing classification systems or as a stand-alone predictive biomarker in instances where gold-standard diagnosis tests are inconclusive or cannot be performed.

The establishment of a cutoff value for MLKL expression levels to categorize patients is critical for clinical applicability. Here, we have shown that splitting the discovery cohort patients into MLKL high and low-expressing categories, through a minimum p-value with bootstrap validation approach, creates two statistically distinct subgroups regarding O.S. We also have shown, using the validation cohort, that the same MLKL cutoff value could be used to assess prognosis in novel patients or different cohorts. One limitation of the present work is that RNAseq is currently too expensive to be used as a standard clinical test, yet it is likely that this technology could reach a more feasible cost in the near future. Other methods to quantitate MLKL expression, such as IHC staining and qPCR, are more affordable and future studies are needed to validate them for this purpose.

The fact that higher MLKL expression is associated with an increased risk of death is quite contrasting with the vast majority of previously published reports, which shows that in several solid tumor types, the upregulation of necroptotic molecules–namely RIPK3 and MLKL–would translate into a better clinical outcome [21, 22, 39–41]. A possible explanation for this effect may be due to the unique immunological traits of the CNS, given that immune responses in this system are much less pronounced and more tightly regulated [42]. Therefore, differently to other tissues, increased necroptotic death and its induced pro-inflammatory response might not lead to a greater effective immune response against the tumor. Instead, it could induce neoangiogenesis, contributing to tumor progression [36, 43]. This association was already shown, for example, in malignant mesothelioma, as the presence of necrotic cancer cells was associated with angiogenesis and worse prognosis [44]. Also, similarly to what was observed for MLKL, we have previously shown that higher RIPK3 expression is associated with worse prognosis in diffuse glioma patients [27], suggesting that increased sensitivity to necroptosis may be also relevant to tumor progression. Indeed, the presence of a necrotic core is still the main parameter to diagnose more aggressive glioma subtypes with much worse prognosis and overall worst clinical outcome [45, 46]. It is not unlikely that at least part of this necrotic core may be due to the higher sensitivity to necroptosis.

In conclusion, MLKL expression strongly associates with glioma patient overall survival, constituting a viable prognostic marker. Contrary to what was previously reported in other solid tumor patients, higher expression of MLKL was observed in patients with worst prognosis and reduced O.S., suggesting a detrimental role of necroptosis in glioma progression. Further studies focused in investigating the role of necroptosis in gliomagenesis and tumor progression will elucidate whether MLKL and RIPK3 are not only relevant LGG prognostic biomarkers but also potential targets for therapeutic intervention, as they constitute the core machinery of necroptosis.

## Supporting information

S1 File Additional variables and regression models. (DOCX)

S2 File External cohort validation. (DOCX)
