# Supplementary material for: Higher Mixed lineage Kinase Domain-like protein (MLKL) is associated with worst overall survival in adult-type diffuse glioma patients
Source: PLoS One. 2023 Aug 31;18(8):e0291019. doi: 10.1371/journal.pone.0291019 (PMC10470898; doi:10.1371/journal.pone.0291019)
Supplement: S1 File — (DOCX) [file pone.0291019.s001.docx]

Supplementary Material I – Additional Variables and Regression Models

**Supplemental Table I:** Description of additional variables available for analysis. The cohort used as discovery set is from the TCGA study ‘Lower Grade Glioma’, available at cBioPortal online platform

| Variables | | Survival Status | | | Total |  |
| --- | --- | --- | --- | --- | --- | --- |
|  |  | Alive | Dead | |  |  |
| Histologic Grade |  | | |  |  | |
| 2 | | 209 (54.1) | 38 (30.2) | | 247 (48.2) |  |
| 3 | | 177 (45.9) | 88 (69.8) | | 265 (51.8) |  |
| Histologic Type | |  |  | |  |  |
| Astrocytoma | | 135 (34.9) | 58 (46.0) | | 193 (37.6) |  |
| Oligoastrocytoma | | 105 (27.1) | 25 (19.8) | | 130 (25.3) |  |
| Oligodendroglioma | | 147 (38.0) | 43 (34.1) | | 190 (37.0) |  |
| Patient Gender | |  |  | |  |  |
| Female | | 173 (44.7) | 55 (43.7) | | 228 (44.4) |  |
| Male | | 214 (55.3) | 71 (56.3) | | 285 (55.6) |  |
| Family History of Cancer | |  |  | |  |  |
| No | | 172 (64.2) | 38 (52.1) | | 210 (61.6) |  |
| Yes | | 96 (35.8) | 35 (47.9) | | 131 (38.4) |  |
| Family History of Primary Brain Tumor | |  |  | |  |  |
| No | | 264 (96.4) | 69 (95.8) | | 333 (96.2) |  |
| Yes | | 10 (3.6) | 3 (4.2) | | 13 (3.8) |  |
| ICD-O-3 Histology Code | |  |  | |  |  |
| 9382/3 | | 104 (26.9) | 27 (21.4) | | 131 (25.5) |  |
| 9400/3 | | 55 (14.2) | 8 (6.3) | | 63 (12.3) |  |
| 9401/3 | | 80 (20.7) | 50 (39.7) | | 130 (25.3) |  |
| 9450/3 | | 93 (24.0) | 18 (14.3) | | 111 (21.6) |  |
| 9451/3 | | 55 (14.2) | 23 (18.3) | | 78 (15.2) |  |
| ICD-O-3 Site Code | |  |  | |  |  |
| C71.0 | | 334 (86.3) | 104 (82.5) | | 438 (85.4) |  |
| C71.1 | | 4 (1.0) | 1 (0.8) | | 5 (1.0) |  |
| C71.2 | | 6 (1.6) | 0 (0.0) | | 6 (1.2) |  |
| C71.3 | | 1 (0.3) | 0 (0.0) | | 1 (0.2) |  |
| C71.4 | | 1 (0.3) | 0 (0.0) | | 1 (0.2) |  |
| C71.9 | | 41 (10.6) | 21 (16.7) | | 62 (12.1) |  |
| History of Ionizing RT to the head | |  |  | |  |  |
| No | | 364 (98.9) | 125 (100.0) | | 489 (99.2) |  |
| Yes | | 4 (1.1) | 0 (0.0) | | 4 (0.8) |  |
| Postoperative Radiotherapy | |  |  | |  |  |
| No | | 100 (49.0) | 20 (34.5) | | 120 (45.8) |  |
| Yes | | 104 (51.0) | 38 (65.5) | | 142 (54.2) |  |
| Neoadjuvant Therapy Prior To Resection | |  |  | |  |  |
| No | | 88 (31.0) | 21 (23.3) | | 109 (29.1) |  |
| Yes | | 196 (69.0) | 69 (76.7) | | 265 (70.9) |  |
| Tumor Relapse/ Progression | |  |  | |  |  |
| No | | 170 (85.4) | 18 (33.3) | | 188 (74.3) |  |
| Yes | | 29 (14.6) | 36 (66.7) | | 65 (25.7) |  |
| Adjuvant Postoperative Targeted Therapy | |  |  | |  |  |
| No | | 100 (48.8) | 25 (43.1) | | 125 (47.5) |  |
| Yes | | 105 (51.2) | 33 (56.9) | | 138 (52.5) |  |
| Preoperative Corticosteroids | |  |  | |  |  |
| No | | 177 (63.2) | 37 (43.5) | | 214 (58.6) |  |
| Yes | | 103 (36.8) | 48 (56.5) | | 151 (41.4) |  |
| Primary Therapy Outcome | |  |  | |  |  |
| Complete Remission/Response | | 78 (43.3) | 5 (9.6) | | 83 (35.8) |  |
| Partial Remission/Response | | 47 (26.1) | 4 (7.7) | | 51 (22.0) |  |
| Progressive Disease | | 13 (7.2) | 26 (50.0) | | 39 (16.8) |  |
| Stable Disease | | 42 (23.3) | 17 (32.7) | | 59 (25.4) |  |
| Prior Cancer Occurrence | |  |  | |  |  |
| No | | 374 (96.6) | 124 (98.4) | | 498 (97.1) |  |
| Yes | | 5 (1.3) | 1 (0.8) | | 6 (1.2) |  |
| Yes, Prior Malignancy | | 7 (1.8) | 1 (0.8) | | 8 (1.6) |  |
| Yes, Bilateral Malignancy | | 1 (0.3) | 0 (0.0) | | 1 (0.2) |  |
| Tumor Site | |  |  | |  |  |
| Posterior Fossa, Brain Stem | | 0 (0.0) | 1 (0.8) | | 1 (0.2) |  |
| Posterior Fossa, Cerebellum | | 2 (0.5) | 0 (0.0) | | 2 (0.4) |  |
| Supratentorial, Frontal Lobe | | 236 (61.0) | 64 (51.2) | | 300 (58.6) |  |
| Supratentorial, NOS | | 6 (1.6) | 2 (1.6) | | 8 (1.6) |  |
| Supratentorial, Occipital Lobe | | 6 (1.6) | 2 (1.6) | | 8 (1.6) |  |
| Supratentorial, Parietal Lobe | | 38 (9.8) | 9 (7.2) | | 47 (9.2) |  |
| Supratentorial, Temporal Lobe | | 99 (25.6) | 47 (37.6) | | 146 (28.5) |  |

N: sample size; ICD-O-3: International Classification of Diseases for Oncology, Third Edition; RT: radiotherapy

**Supplemental Table II:** Univariate model of additional explanatory variables.

|  | N | HR | 95% CI for HR | | p-value |
| --- | --- | --- | --- | --- | --- |
|  |  |  | LL | UL |  |
| Histologic Grade and Type |  |  |  |  |  |
| Oligodendroglioma 2 | 110 | Ref. |  |  |  |
| Oligoastrocytoma 2 | 74 | 1.060 | 0.491 | 2.290 | 0.882 |
| Astrocytoma 2 | 62 | 0.963 | 0.419 | 2.213 | 0.929 |
| Oligodendroglioma 3 | 79 | 2.596 | 1.398 | 4.820 | **0.003** |
| Oligoastrocytoma 3 | 55 | 2.729 | 1.374 | 5.417 | **0.004** |
| Astrocytoma 3 | 131 | 4.112 | 2.393 | 7.065 | **<0.001** |
| Family History of Cancer |  |  |  |  |  |
| No | 209 | Ref. |  |  |  |
| Yes | 131 | 1.566 | 0.986 | 2.489 | 0.058 |
| Postoperative Radiotherapy |  |  |  |  |  |
| No | 120 | Ref. |  |  |  |
| Yes | 142 | 2.039 | 1.179 | 3.527 | **0.011** |
| Neoadjuvant Therapy Prior To Resection |  |  |  |  |  |
| No | 109 | Ref. |  |  |  |
| Yes | 265 | 1.323 | 0.802 | 2.183 | 0.272 |
| Tumor Relapse/ Progression |  |  |  |  |  |
| No | 188 | Ref. |  |  |  |
| Yes | 65 | 2.850 | 1.574 | 5.160 | **0.001** |
| Adjuvant Postoperative Targeted Therapy |  |  |  |  |  |
| No | 125 | Ref. |  |  |  |
| Yes | 138 | 1.791 | 1.039 | 3.087 | **0.036** |
| Preoperative Corticosteroids |  |  |  |  |  |
| No | 213 | Ref. |  |  |  |
| Yes | 151 | 1.541 | 0.991 | 2.397 | 0.055 |
| N: sample size; HR: Hazard ratio; LL: Lower limit; UL: Upper limit; CI: Confidence Interval; | | | | | |

**Supplemental Table III:** Multivariate model of MLKL expression controlled by histological grade/type and age at diagnosis.

| Variables | N (%) | HR | 95% CI for HR | | p-value |
| --- | --- | --- | --- | --- | --- |
|  |  |  | LL | UL |  |
| Histologic type and grade | |  |  |  |  |
| Oligodendroglioma 2 | 110 (21.6) | Ref. |  |  |  |
| Oligoastrocytoma 2 | 74 (14.5) | 1.51 | 0.69 | 3.31 | 0.299 |
| Astrocytoma 2 | 62 (12.2) | 1.32 | 0.57 | 3.08 | 0.508 |
| Oligodendroglioma 3 | 79 (15.5) | 2.08 | 1.11 | 3.93 | 0.023 |
| Oligoastrocytoma 3 | 55 (10.8) | 3.12 | 1.57 | 6.24 | 0.001 |
| Astrocytoma 3 | 131(25.4) | 4.25 | 2.43 | 7.44 | <0.001 |
| MLKL |  |  |  |  |  |
| Low | 400 (78.3) | Ref. |  |  |  |
| High | 111 (21.7) | 1.87 | 1.25 | 2.79 | 0.002 |
| Diagnostic age | 511 (100) | 1.06 | 1.04 | 1.08 | <0.001 |

N: sample size; HR: Hazard ratio; LL: Lower limit; UL: Upper limit; CI: Confidence Interval; Ref.: Reference.
