# Supplementary material for: Higher Mixed lineage Kinase Domain-like protein (MLKL) is associated with worst overall survival in adult-type diffuse glioma patients
Source: PLoS One. 2023 Aug 31;18(8):e0291019. doi: 10.1371/journal.pone.0291019 (PMC10470898; doi:10.1371/journal.pone.0291019)
Supplement: S2 File — (DOCX) [file pone.0291019.s002.docx]

Supplementary Material II – External Cohort Validation

**Supplemental Table IV:** Description of the main variables available for analysis in the validation cohort. The cohort used as validation set is from the CGGA

| Variable | O.S. Status | | Total |
| --- | --- | --- | --- |
|  | Alive | Dead |  |
| MLKL |  |  |  |
| Median  (1ºQ; 3ºQ) | -0.6  (-0.79; -0.29) | -0.41  (-0.79; 0.01) | -0.55  (-0.79; -0.19) |
| Age at Diagnosis |  |  |  |
| Median  (1ºQ; 3ºQ) | 39  (34; 44) | 46  (36; 56) | 40  (35;46) |
| IDH Mutation |  |  |  |
| No | 20 (20%) | 18 (53%) | 43 (30%) |
| Yes | 80 (80%) | 16 (47%) | 98 (70%) |

Numerical data described as non-parametrical data, considering median and interquartile range. Categorical data described with number of samples and percentile. Q: Quartile

**Supplemental Table V:** Univariate model of the main explanatory variables in the validation cohort. See other models in Table S 3.

| Variables | N (%) | HR (95% IC) | p-value |
| --- | --- | --- | --- |
|  |  |  |  |
| MLKL* | 134 | 1.09 (1.04, 1.14) | <0.001 |
| Age at diagnosis | 134 | 1.07 (1.04, 1.11) | <0.001 |
| IDH Mutation |  |  |  |
| Yes | 96 (0.72) | Ref. |  |
| No | 38 (0.28) | 4.88 (2.47, 9.61) | <0.001 |

N: sample size; HR: Hazard ratio; CI: Confidence Interval; *Consider 0.1 as unit of variation in the z-score scale for interpretation of MLKL HR estimation.

**Supplemental Table VI:** Univariate model for MLKL expression levels in the validation cohort. Groups were split considering the optimal cutoff value for the specified gene in the discovery cohort.

|  | N (%) | HR (95% IC) | p-value |
| --- | --- | --- | --- |
| MLKL |  |  |  |
| Low | 78 (58) | Ref. |  |
| High | 56 (42) | 2.64* (1.32, 5.27) | 0.006** |

N: sample size; HR: Hazard ratio; CI: Confidence Interval; MLKL samples with a z-score of ≤0.439 were pooled in the low expression group, whereas samples with >0.439 were pooled in the high expression group. *Hazard Ratio corrected by heuristic contraction factor; **p-value corrected by Lausen & Schumacher method.


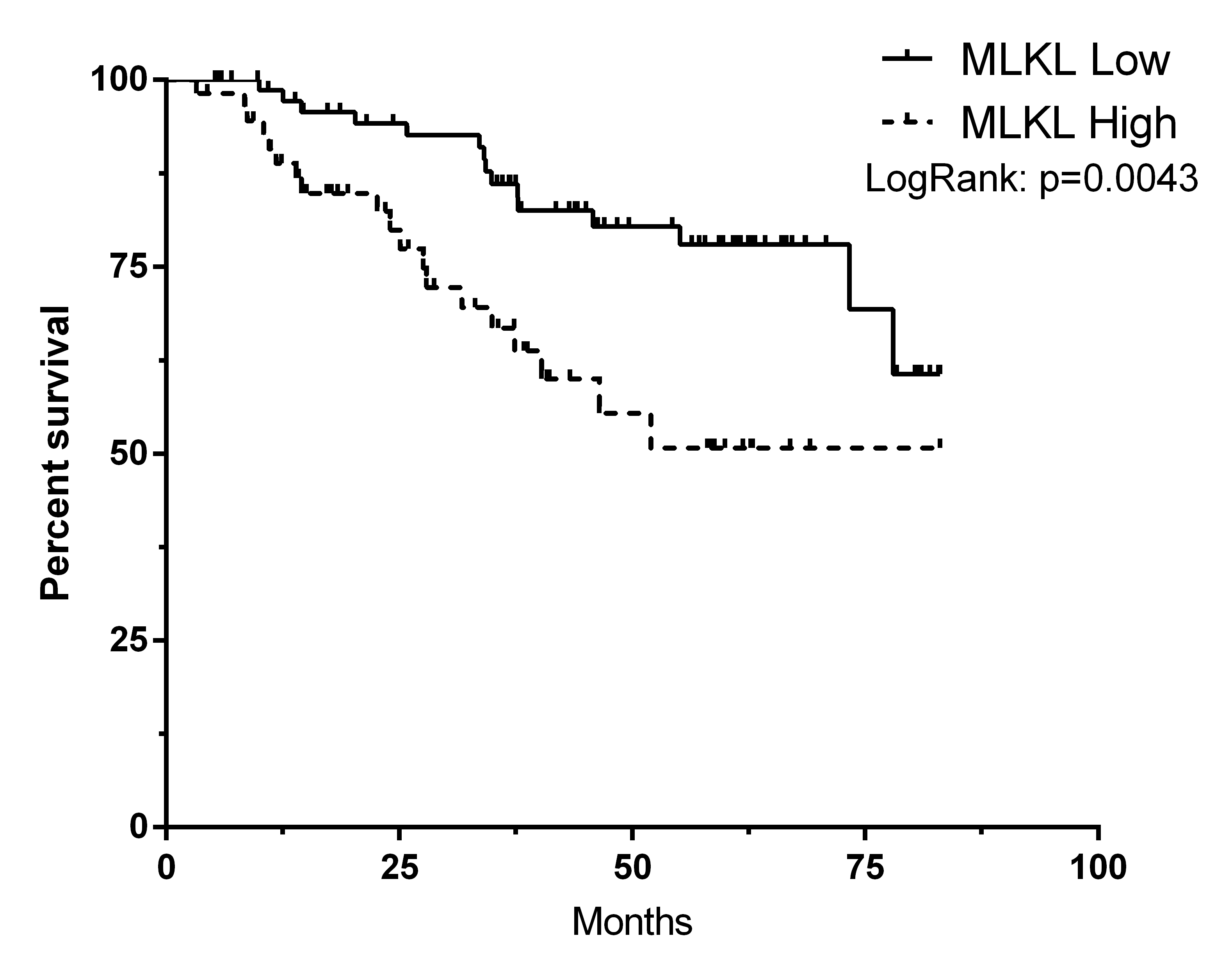


**Supplemental Figure I:** Kaplan Meier survival analysis of LGG validation patients considering the expression of MLKL. We grouped patients into high (red) or low (blue) expression levels, according to the optimal cut-off value estimated in the discovery cohort. For inferential interpretation, we performed LogRank tests.


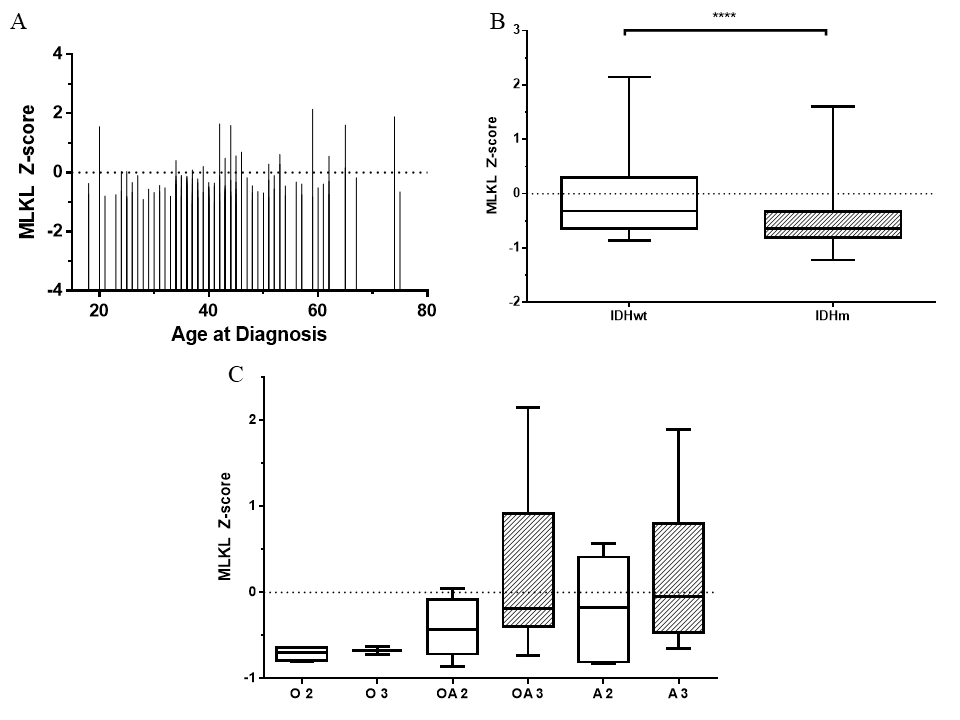


**Supplemental Figure II:** Assessment of MLKL’s association with known clinical variables in the validation cohort. Using continuous data of MLKL’s expression, we checked for (A) correlation between MLKL and Age at diagnosis and association between MLKL expression and both (B) main prognostic marker IDH mutational status and (C) histological diagnostic

**Supplemental Table VII:** Multivariate model of MLKL expression controlled by IDH mutation/ and age at diagnosis in the validation cohort.

| Variables | N | HR | 95% CI for HR | | p-value |
| --- | --- | --- | --- | --- | --- |
|  |  |  | LL | UL |  |
| IDH1 mutation |  |  |  |  |  |
| Yes | 96 | Ref. |  |  |  |
| No | 38 | 3.92 | 1.94 | 7.92 | <0.001 |
| MLKL |  |  |  |  |  |
| Low | 78 | Ref. |  |  |  |
| High | 56 | 1.96 | 0.96 | 4.02 | 0.066 |
| Age at diagnosis | 134 | 1.05 | 1;02 | 1.09 | 0.004 |

N: sample size; HR: Hazard ratio; CI: Confidence Interval; LL: Lower Limit; UL: Upper Limit; Ref.: Reference.
